# Endoscopy-assisted high anterior cervical approach in craniovertebral junction (CVJ)

**DOI:** 10.3389/fsurg.2022.984015

**Published:** 2022-10-31

**Authors:** Pengfei Li, Kaixuan Wang, Hongming Ji, Gangli Zhang, Shengli Chen, Shiyuan Zhang, Ian F. Dunn, Changchen Hu

**Affiliations:** ^1^ Department of Neurosurgery, Shanxi Provincial People's Hospital, Shanxi Medical University, Taiyuan, China; ^2^ Department of Neurosurgery, University of Oklahoma Health Sciences Center, Oklahoma City, OK, United States; ^3^ Department of Neurosurgery, Shuozhou People's Hospital, Shuozhou, China

**Keywords:** endoscope-assisted surgery, high anterior cervical approach, craniovertebral junction, endoscopic technique, application

## Abstract

**Background:**

Surgical procedures in the craniovertebral junction (CVJ) suffer from specific challenges due to the proximity between the cranium and spine containing the critical neurovascular structures and the brainstem, respectively. Owing to the complex transitional zone, it is highly challenging for classic surgical approaches to practically acquire the additional exposure to neurovascular structures of the CVJ. Inspired by these facts, we explore the feasibility of an endoscopy-assisted high anterior cervical approach in the CVJ.

**Methods:**

To explore the feasibility of an endoscopy-assisted approach, we quantitatively assessed the surgical corridor and extent of exposure of the CVJ in 6 cadaveric specimens using 0° and 30° endoscopes.

**Results:**

The applied endoscopes provided adequate exposure to neurovascular structures and the brainstem in the CVJ. Notably, the resection of the anterior arch of C1 is avoided in minimal anterior clivectomy. Further, improved exposure of the CVJ is obtained after removing the odontoid.

**Conclusion:**

An endoscope-assisted high anterior cervical approach in the CVJ significantly preserved the cervical spine stability while minimalizing the risk of neurovascular injury within the surgical corridor.

## Background

The craniovertebral junction (CVJ) characterizes the complex transitional zone between the cranium and the spine. The surgical procedures in the CVJ present specific challenges due to the proximity of critical neurovascular structures and the brainstem. In this context, several ventral and dorsal approaches to operating CVJ have been reported for accessing a variety of pathologies. These approaches are classified into anterior, posterior, and lateral strategies. In this regard, several classic approaches, such as the midline suboccipital and the transcondylar approaches, are preferred for operating posterior and lateral lesions at the cervicomedullary junction (1). To this end, the ventral lesions, especially extradural pathologies, could be readily accessed through the anterior corridors, including transoral and transnasal methods (2–7). Notably, it is highly challenging to apply transoral and transnasal approaches in the setting of oral or nasal cavity lesions or when the majority of the lesion extends laterally or caudally into the cervical spine. Previous reports indicated that the high anterior cervical approach provided adequate decompression of the cervicomedullary junction (8). In addition, several reports showed that anterior cervical fixation or fusion could be performed through this approach (9). Simultaneously, the oropharyngeal mucosa could be preserved with fewer pharyngeal complications. However, this approach suffers from a significant shortcoming of very long working distance under the microscope, which utilizes the posterior pharyngeal space. In addition, it is difficult to retract and gain access to the posterior pharyngeal wall soft tissue due to maxilla and muscle soft tissue constraints. Moreover, there exists only a tiny bony window through the clivus within minimal anterior clivectomy (8). Owing to these shortcomings, it is practically challenging to obtain extra exposure to neurovascular structures in the CVJ. Recently, endoscopes have garnered increasing attention from researchers as a part of the neurosurgical armamentarium. Inspired by these aspects, herein, we explored the feasibility of the endoscope-assisted high anterior cervical approach for extended exposure in the CVJ.

## Methods

All anatomical dissections were executed at the Skull Base Laboratory and Minimally Invasive Neurosurgery Laboratory. The embalmed human cadaveric heads (*n* = 6) from body donations were obtained from the anatomical laboratory of Shanxi Medical University, Shanxi, China. Notably, the donors were informed and agreed that cadavers would be used for medical research. The study was approved by the Ethical Committee of the Hospital, and required permissions were obtained to utilize these samples from Shanxi medical university. The microscopic anatomical dissections were performed under 3–40x optical magnifications using an operating microscope (Global Instruments, Trenton, MO, USA). To this end, the endonasal anatomical dissections were carried out using 0° and 30° rod-lens, 4-mm diameter, 18-cm length, Hopkins II endoscopes (Karl Storz Endoscopy, Tuttlingen, Germany). These endoscopes were connected to a high-definition camera and projected onto a monitor. All the digitally recorded data were stored in a workstation for future reference (Gefen System, Petaluma, CA, USA).

## Results

### Surgical procedure

**Figure 1:**
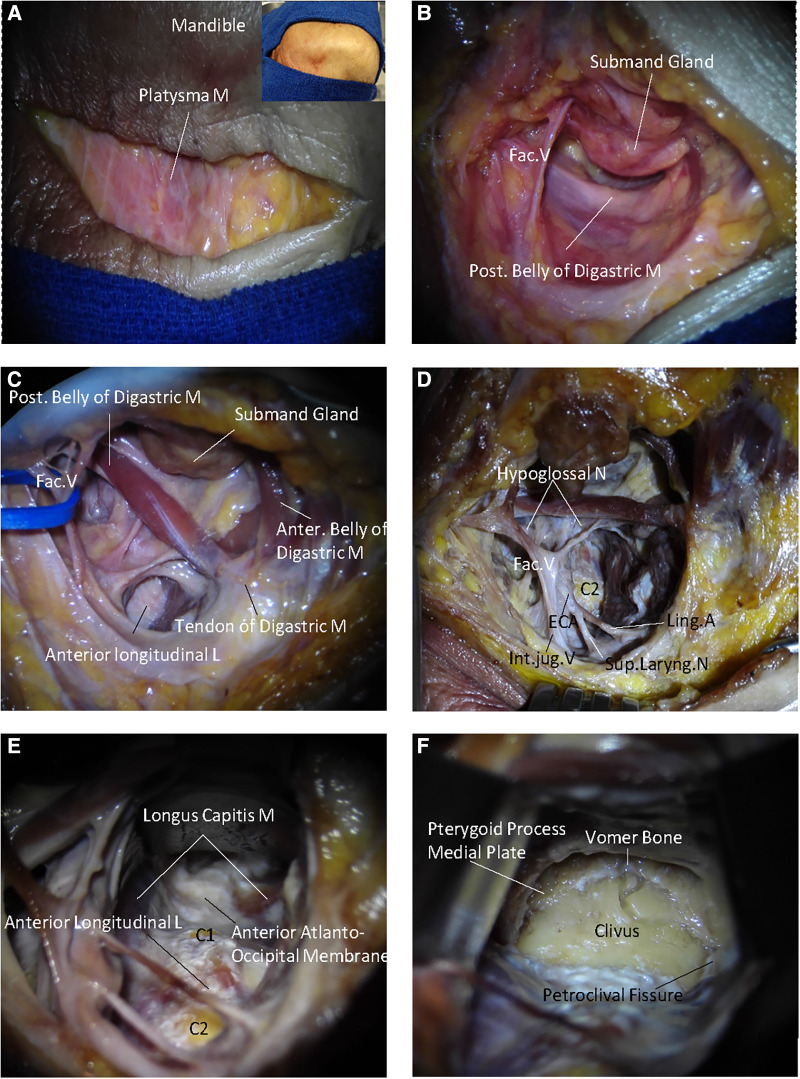
The surgical procedure of endoscope-assisted high anterior cervical approach. (**A**) A straight skin incision is made about 2 cm below and parallel to the inferior border of the mandible. (**B**) The platysma muscle is elevated. The submandibular gland and posterior belly of the digastric muscle are exposed. (**C**) The submandibular gland and posterior belly of the digastric muscle are released and retracted and the entrance of the retropharyngeal space is exposed. (**D**) The hypoglossal nerve passes and the lingual artery is divided; and the retropharyngeal space is opened. (**E**) The pharyngeal muscles are retracted medially and further separated deeply. The anterior surface of the cervical vertebrae and anterior atlantooccipital membrane are exposed. (**F**) The lower clivus is exposed, including the vomer, pterygoid precess medial plate, petroclival, and the anterior rim of the foramen magnum. The arrows indicate the direction in the space, S represents the superior part of the cadaveric heads, M represents the medial part of the cadaveric heads.

The high anterior cervical retropharyngeal approach to the upper cervical spine has been described in detail (10–12). In a case, Russo and colleagues applied this approach to the clivus and foramen magnum (8). In this study, this approach was executed following the series of steps discussed below. The procedure was performed with the patient positioned supine. Initially, the patient's head was extended 20°–30° and rotated 30°–45° away from the side. In addition, the mandible was displaced superiorly. Further, an incision was made approximately 3–4 cm inferior and parallel to the mandible, avoiding injury to the marginal mandibular branch of the facial nerve (Figure 1A). Then, the platysma was divided and retracted superiorly, thus exposing and elevating the submandibular gland (Figure 1B). Further, the posterior belly of the digastric muscle was brought into view under the submandibular glands. Then, the anterior belly of the digastric muscle was retracted medially, as well as the facial artery and vein laterally. The posterior belly and tendons of the digastric muscle were elevated superiorly. After dividing the posterior belly deep, the hypoglossal nerve was revealed, passing inferior to the muscle (Figures 1C,D). The hypoglossal nerve was carefully dissected and retracted rostrally. The external carotid artery and the facial artery branch were then retracted laterally and superiorly, and the lingual artery was retracted inferiorly (Figures 1B,C). The pharyngeal muscles were then retracted medially, thus opening the retropharyngeal space. The pharyngeal muscles were further separated deeply, and the anterior tubercle of C1, as well as the anterior surface of the cervical vertebrae, were exposed (Figure 1E). Subsequently, the prevertebral fascia and the anterior longitudinal ligament in the midline were preserved, which are important for cervical spine stability, exposing the entire arch of C1 and the body of C2. The anterior atlantooccipital membrane and the longus capitis muscles were detached from the anterior rim of the foramen magnum and midlateral portion of the clivus (Figure 1F). Accordingly, the upper boundary was the vomer and pterygoid process medial plate, and the petroclival fissure as the bilateral boundary.

### Anatomical measurements

**Figure 2:**
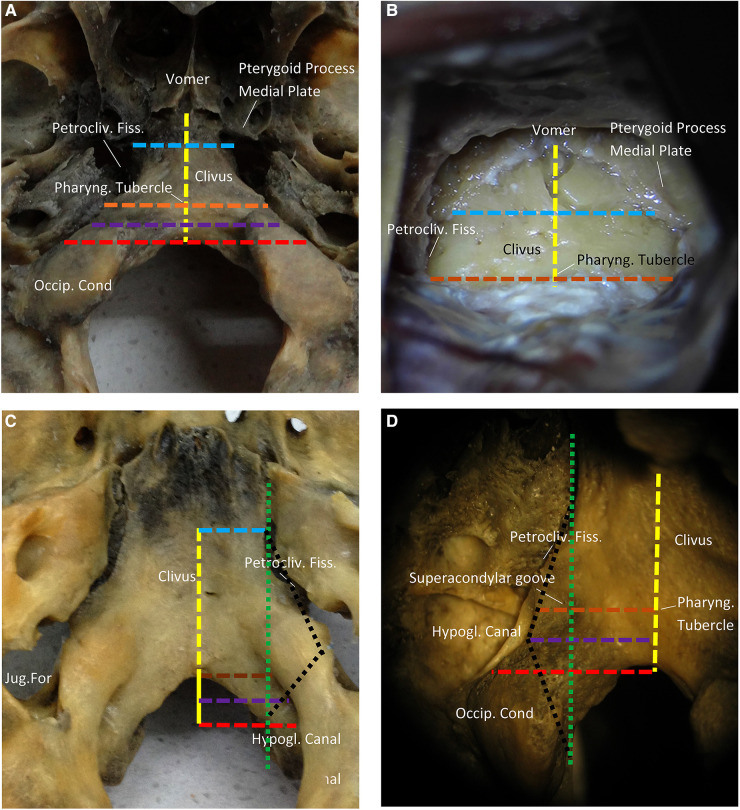
Surgical anatomy and measurements of the lower clivus and ventral foramen magnum. (**A**) The ventral side of the clivus. Occipital foramen-vomer (yellow line). The width of the clivus at the pharyngeal tubercle level (brown line). The width of clivus at the pterygoid process medial plate level (blue line). The width of clivus at the hypoglossal canal outside the hole midpoint (purple line). The width of the clivus at the inferior margin of the hypoglossal canal outside the hole (red line). (**B**) Initial exposure of the clivus. (**C**) The interior side of the clivus. The green line is the line from the petroclival fissure to the hypoglossal canal inside the hole. The lateral limit of the medial condylectomy is the hypoglossal canal inside the hole. (**D**) The hypoglossal canal outside the hole. It is directed backward and medially at a 45° angle with the sagittal plane. The pharyngeal tubercle is at the same level with the superior margin of hypoglossal canal outside the hole. The lateral limit of the medial condylectomy is defined by the dotted black line (petroclival fissure to superior margin hypoglossal canal outside the hole to the hypoglossal canal inside the hole).

Further, the anatomical structures of the free clivus in the specimen were measured. The mean distance from the anterior rim of the foramen magnum to the vomer was measured as 27.1 mm (range 26–28.9 mm). The mean width of the clivus at the pterygoid process medial plate level was around 21.6 mm (range 21.0–22.3 mm). The mean width of the clivus at the pharyngeal tubercle level was 27.8 mm (range 26.9–28.4 mm). The mean width of the clivus at the hypoglossal canal outside the hole midpoint level was 28.7 mm (range 27.6–29.1 mm). The mean width of the clivus at the inferior margin of the hypoglossal canal outside the hole was around 29.6 mm (range 28.7–31.1 mm, Figure 2A).

In addition, we measured the mean distance of the previous step on the heads, which showed an excellent exposure of the clivus. The anterior rim of the foramen magnum has adhered to the longus capitis muscle, rectus capitis anterior muscle, pharyngobasilar fascia, and mucosa. It should be noted that dissecting from the anterior margin of the foramen magnum was challenging, especially the fascia on the supracondylar groove. In addition, it was challenging to dissect from the cortical bone surface of the groove, which was an essential landmark on the clivus for localizing the hypoglossal canal due to similar depths. At this stage, the mean distance from the pharyngeal tubercle to the vomer was measured as 19.1 mm (range 18.7–20.9 mm). The mean width of the clivus at the pterygoid process medial plate level was recorded as 20.1 mm (range 19.3–20.9 mm). The mean width of the clivus at the pharyngeal tubercle level was around 25.9 mm (range 24.9–26.4 mm, Figure 2B). Nevertheless, no noticeable difference was observed compared with the measurements of the free clivus, indicating the convenience of separating the mucosa of the clivus bilaterally from the petroclival fissure.

In the case of CVJ, the hypoglossal canal and nerve are essential structures. The hypoglossal canal was directed posteriorly and medially at a 45° angle with the sagittal plane, thus locating its extracranial outside hole proximately above the junction of the anterior and middle third of the occipital condyle, as well as medial to the jugular foramen. The interior view of the clivus indicated that the hypoglossal canal was positioned at the back of the free anterior rim of the foramen magnum. Thus, the medial border of the intracranial hole and the petroclival fissure possessed the same vertical line (Figure 2C). In the exterior view of the clivus, the hypoglossal canal was located at the front of the free anterior rim of the foramen magnum. It should be noted that the midpoint of the hypoglossal canal outside the hole acted as the lateral limiting point. Accordingly, a black line was dotted as the lateral limit of the medial condylectomy (petroclival fissure to the midpoint of the hypoglossal canal outside the hole to the hypoglossal canal inside the hole, Figure 2D).

### Exposure of CVJ

**Figure 3:**
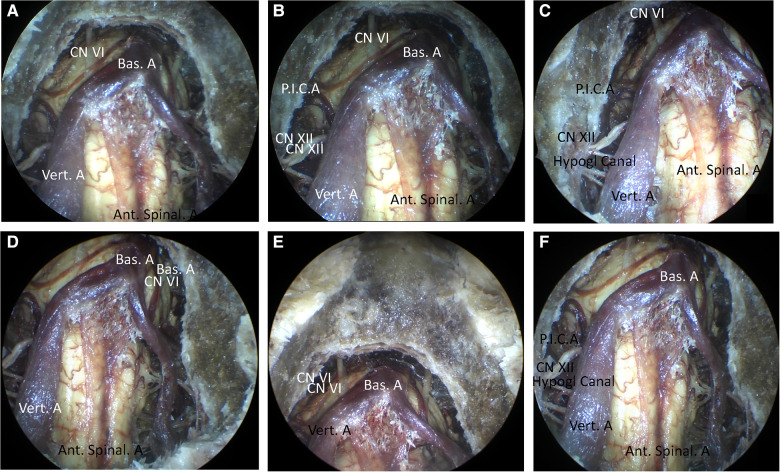
(**A**) The view of the resection of the clivus (0°). (**B–F**) The view of the resection of the clivus and the atlas. (**B**) The view of resection of the clivus and the atlas (0°). (**C**) The view of the resection of the clivus and the atlas (30°, right). (**D**) The view of the resection of the clivus and the atlas (30°, left). (**E**) The view of the resection of the clivus and the atlas (30°, cranial). (**F**) The view of the resection of the clivus and the atlas (30°, caudal).

The exposure of the CVJ was performed by disclosing the clivus and the high cervical area. Initially, the clivus resection was performed using a high-speed drill or rongeur through the midline of the inferior portion of the clivus. Indeed, the upper boundary, i.e., the sphenoidal sinus, was limited laterally by the medial border of the petroclival fissure. In contrast, the lower boundary was the anterior rim of the foramen magnum. In this resection, the atlantooccipital anterior membrane, anterior longitudinal ligament, and apical ligament of the dens of C1 were preserved. However, there existed a 20 mm × 30 mm bony window through the clivus, as previously reported by Russo and colleagues (8). After opening the dura mater, the ventral aspect of the brainstem and the related vascular and neural structures could be observed (Figure 3A). The proximal segment of the basilar artery (BA) and bilateral vertebral artery (VA) could be initially detected. From the top view, the 6th cranial nerve (abducent nerve) could be observed from its origin. The abducent nerve is very very important when performing the approach. The abducens nerve runs in the prepontine cistern between the clivus and the pontine base, and the hypoglossal and oculomotor nerves are adjacent to the rostral and caudal ends of the clivus, respectively. The glossopharyngeal, vagus and facial auditory nerves all run lateral to the clivus. in the brain cistern. There is a potential risk of injuring the abducens nerve, when endoscopically enlarged transnasal approach for the treatment of clivus tumors. This nerve root enters and exits the brainstem zone (REZ) and cisternal segment (CS), which are particularly vulnerable to clivus resection and dura dissection *via* the clivus approach. The Eustachian tube is a constant and easily identifiable structure, and most EEA transclivi approach does not necessarily require resection of ET, so ET can be used as an anatomical landmark for CN VI in endoscopic skull base surgery *via* transclivi approach. The pontomedullary sulcus was exposed in the middle. The anterior spinal artery could be observed in the midline, while the posterior inferior cerebellar artery (PICA) and cranial nerve (CN) XII could be partly observed bilaterally.

Further, the endoscope-assisted high anterior cervical approach was executed by expanding the bone window. The anterior atlantooccipital membrane, and anterior longitudinal ligament were preserved. After drilling the medial third of the lateral mass of C1 and the anteromedial third of the occipital condyle, the CVJ vascular and neural structures were observed using 0° and 30° rod-lens endoscopes. In addition, the BA, VA, abducens nerve, and pontomedullary sulcus could be observed, increasing the exposure of the inferior field. The hypoglossal canal, CN XII, and P.I.C.A. were still bilaterally observed partially using 0° endoscopes, with an improved observation range (Figure 3B). However, it could be observed completely using 30° endoscopes (Figures 3C–F). Notably, the occipital condyle anatomic levels, in turn, were observed as cortical bone, soft cancellous bone, hard cortical bone, and hypoglossal canal. Moreover, it should be noted that the deeper layer of hard cortical bone of the occipital condyle should be carefully drilled to protect the hypoglossal nerve.

**Figure 4:**
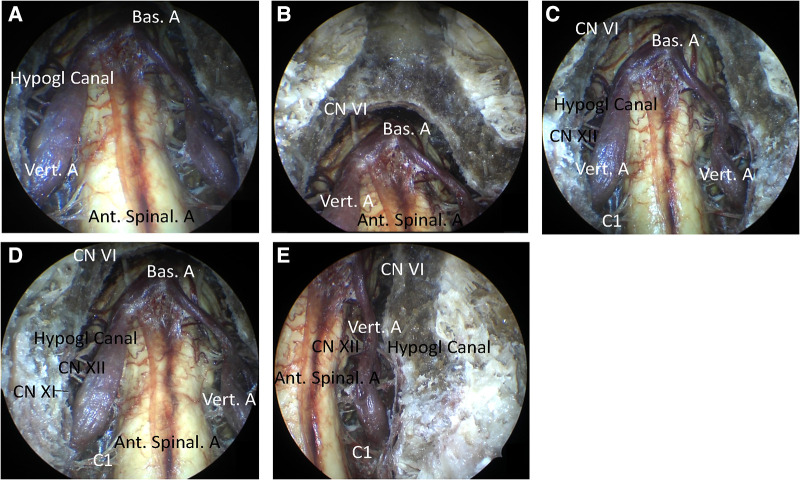
The view of the resection of the clivus, atlas, and dens. (**A**) The view of the 0° endoscope. (**B**) The view of the 30° endoscope (cranial). (**C**) The view of the 30° endoscope (caudal). (**D**) The view of the 30° endoscope (right). (**E**) The view of the 30° endoscope (left).

Further, drilling down to the level of the body of C2 was continued to expand the inferior field exposure. After opening the dura mater, the cervicomedullary junction was then exposed. Notably, the CVJ area was sufficiently exposed after drilling out the dens (Figure 4A). In addition, other structures such as BA, VA, abducens nerve, pontomedullary sulcus, hypoglossal canal, CN XII, and PICA were exposed. Further, the vascular and neural structures of the expanded CVJ region were observed using a 0° endoscope along with the upper cervical spinal cord. In addition, the vertebral artery intradural segments were observed completely, specifically from their dural entrance points to the supramedial rising segment. More importantly, the spinal nerve C1 could be bilaterally observed completely using a 0° endoscope. Together, using a 30°endoscope resulted in greater exposure of the CVJ region than a 0° endoscope (Figures 4B–E).

### Exposure degree and resection range of the CVJ

**Figure 5:**
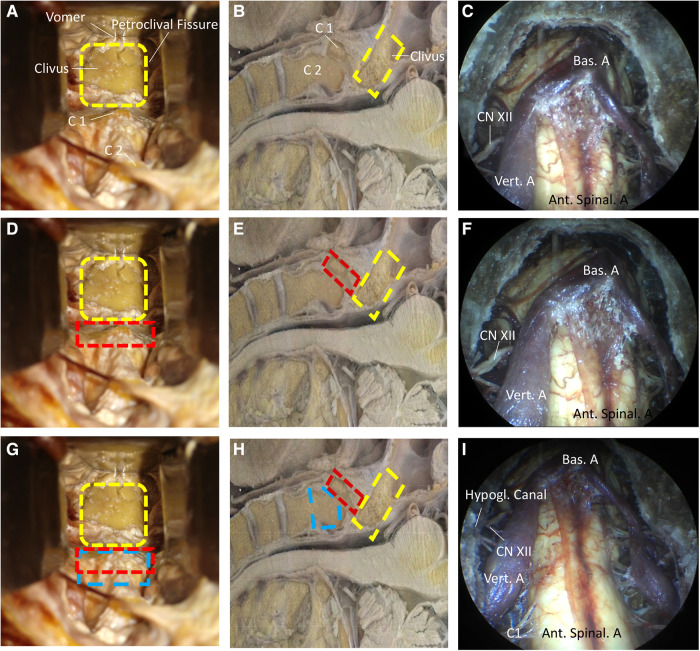
The relation of exposure degree and resection range of CVJ. (**A,D,G**) The different resection range of CVJ. (**B,E,H**) From the sagittal display, different degrees of resection of CVJ in another specimen. (**C,F,I**) Exposure degree under the endoscope. (**A–C**) The view of the resection of the clivus. The orange portion represents the resected clivus. (**D–F**) The view of the resection of the clivus and the atlas. The green portion represents the resected atlas. (**G–I**) The view of the resection of the clivus, atlas, and odontoid. The yellow portion represents the resected odontoid. It is important to resect the odontoid in order to sufficiently expose the CVJ in the anterior cervical approach to the CVJ. There is not an obvious increase in the exposure degree of the CVJ when the atlas is drilled out based on the resected clivus in the anterior cervical approach. After removing the odontoid. A greater exposure of the CVJ area is obtained. It is imperative to resect the dens in order to sufficiently expose the CVJ in the anterior cervical approach. The arrows indicate the direction in the space, S represents the superior part of the cadaveric heads, M represents the medial part of the cadaveric heads.

The relationship between the exposure degree and resection range of the CVJ is summarized in Figure 5. Figures 5A–C display the endoscopic views of the resected clivus in the endoscope-assisted high anterior cervical approach, while Figures 5D–F display endoscopic views of resectioning the clivus and atlas. It was observed from the results that there existed no apparent increase in the exposure degree of the CVJ. Figures 5G–I display the endoscopic views of the resectioned clivus, atlas, and odontoid. The experimental results indicated that no apparent increase in the exposure degree of the CVJ was observed during drilling out the atlasbased on the resected clivus in the anterior cervical approach (Figures 5C,F). After removing the odontoid, greater exposure to the CVJ area was obtained (Figures 5C,F,I). Together, these findings indicated that it was imperative to resect the dens to sufficiently expose the CVJ in the anterior cervical approach, especially for large lesions involving the high cervical spinal region.

## Discussion

**Figure 6:**
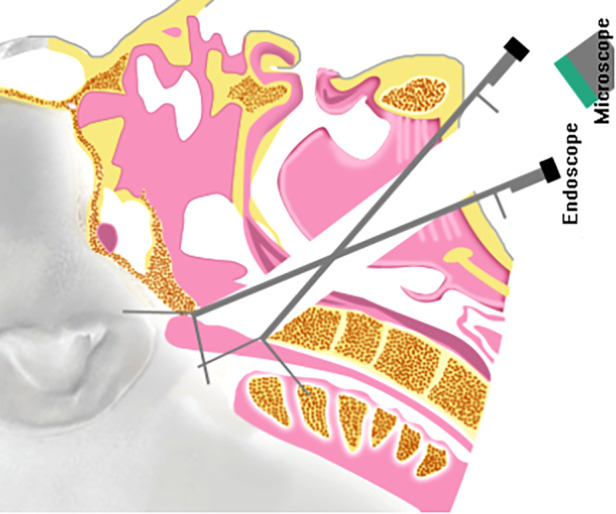
The comparison of the high anterior cervical approach to the craniovertebral junction under the endoscope and microscope.

Indeed, some recent studies have reported the applicability of the high anterior cervical approach. In a case, Vender and colleagues demonstrated anterior cervical fixation or fusion through the endoscopic approach (9). In another case, Park and coworkers studied the high anterior cervical approach for the upper cervical spine fixation (13). In addition, Singh et al. reviewed the high anterior cervical retropharyngeal approach in ventral surgical approaches to CVJ chordomas (14). Russo and colleagues demonstrated a microsurgical anatomy study of the high anterior cervical approach to the clivus and foramen magnum (8). Although a deep retractor and a self-retaining retractor could be employed to elevate the pharyngeal mucosa and maintain lateral displacement of the longus capitis, rectus capitis anterior, and longus colli, the working distance could remain long under the microscope through this approach. It was highly challenging to retract the posterior pharyngeal wall soft tissue due to the maxilla and muscle soft tissue constraints. In this study, the ventral surfaces of the foramen magnum, clivus, petroclival region, and CVJ were exposed under the microscope in a tubular surgical field (Figure 6). In addition, there was only a 20 mm × 30 mm bony window through the clivus within minimal anterior clivectomy (8). Considering these aspects, it could be challenging to obtain extra exposure to the neurovascular structures of the CVJ practically. These factors significantly limited the application of this approach in clinical practice. To this end, a fish eye effect under endoscopy could be utilized to obtain a multi-angle and close-distance observation of the operative region (Figure 6). This approach could substantially overcome the limitation of the tubular surgical field under the microscope. In addition, endoscope technology as part of the neurosurgical armamentarium has recently gained increasing attention from researchers. Considering these aspects, in this study, we demonstrate the development of the endoscope-assisted high anterior cervical approach.

While utilizing a deep retractor and a self-retaining retractor to expose the CVJ, the visualization of the inferior portion of the clivus and the anterior rim of the foramen magnum was obstructed by the anterior arch of C1 under the microscope during the high anterior cervical approach (8). At this stage, the resection of the anterior arch of C1 between the lateral masses was converted to a normal drilling or resection step, even in minimal anterior clivectomy (8). The deep retractor was repeatedly pushed towards the bilateral and upward sides to obtain greater exposure *via* the operation corridor under the microscope. Nonetheless, it should be noted that the blood vessels and nerves of the neck could be damaged easily. Utilizing the multi-angle and close distance observation of the endoscope, the inferior portion of the clivus and the anterior rim of the foramen magnum could be observed (Figure 4), avoiding the resection of the anterior arch of C1 and maintaining the stability of the cervical spine.

Different descriptions of the lateral limit of resection have been reported in the literature. In a case, Wang et al. designed the vertical line (from the foramen lacerum to the occipital condyle) in medial condylectomy as the lateral limit of the medial condylectomy (15). In another case, Russo et al. described the lateral limit of bone resection, the petroclival fissure, the anteromedial third of the occipital condyle, and the anterior half of the jugular tubercle (8). Notably, all these landmarks existed in the free occipital bone. In the actual operation, these landmarks were covered or embedded with other tissue, leading to challenges in judgment during the operation itself. Similarly, the same lateral limit of resection was encountered. However, we believed that the intraoperative assessment should be simple.

The petroclival fissure and the medial border of the intracranial hole represented the superolateral and inferior-lateral limits of resection. In the interior view of the clivus, the medial border of the intracranial hole and the petroclival fissure possessed the same vertical lines (Figure 2C). The midpoint of the hypoglossal canal outside the hole acted as the limiting lateral point (Figure 2D). It was observed that the supracondylar groove and hypoglossal canal were at the same craniocaudal level, and the hypoglossal canal was deep enough. As a surface landmark on the clivus for localizing the hypoglossal canal, thus, the lateral point of the supracondylar groove could act as the lateral limit during the operation (Figures 2C,D). Therefore, we could outline the boundaries for resection (black dot line) using the petroclival fissure, vertical line, and the lateral point of the supracondylar groove during the operation. All of these landmarks were marked on the outside surface of the occipital bone.

The high anterior cervical approach is often used in the upper cervical spine, such as for chordomas of the upper cervical spine (16), fusion at the cervical spine (9), cervical pyogenic C1–2 abscess (17), and cervical spondylosis (18). On the one hand, there existed a degree of angle between the spine's long axis and the operation corridor in the high anterior cervical approach, in some instances, an acute angle, less than 45°. As shown in Figure 6, it was relatively easier to operate upper cervical spine lesions. While, in the cases of the lesions located in the lower clivus and foramen magnum, the degree between the spine's long axis and the operation corridor could be more negligible. On the other hand, it could be difficult to resect the anterior atlantooccipital membrane, occipital condyle, atlantooccipital joint, and apical and alar ligaments.

**Figure 7:**
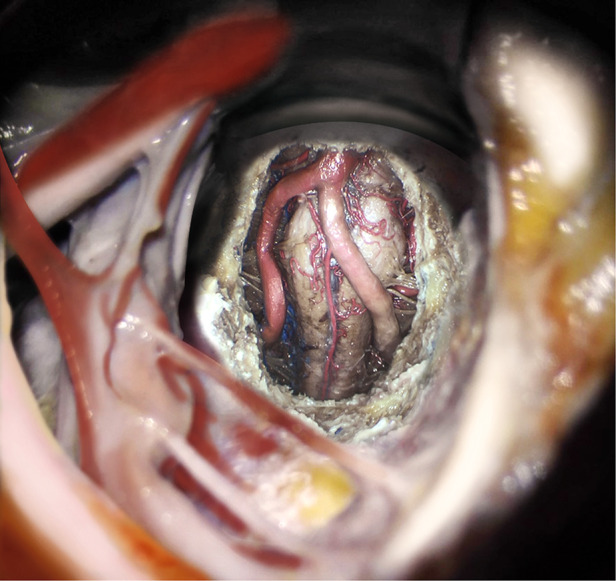
Artist's illustration of the exposure of the high anterior cervical approach to the craniovertebral junction.

Furthermore, the upper structures in the bone windows could be observed due to the acute angle between the operation corridor and the operation bone window (Figure 7). When the atlas was drilled out (Figures 5D,E), no significant increase in the acute angle between the operation corridor and operation bone window was detected. Thus, no evident increase in exposure degree of the CVJ was observed compared with the clivectomy in the anterior cervical approach (Figures 5C,F). After removing the odontoid, greater exposure of the CVJ area was observed (Figures 5C,F,I). Together, these findings indicated that it was imperative to resect the dens to sufficiently expose the CVJ in the anterior cervical approach, especially for large lesions involving the high cervical spinal region.

## Conclusion

In summary, the applicability of the endoscopy-assisted high anterior cervical approach in the CVJ has shown excellent feasibility. This approach maintained the stability of the cervical spine and minimized the chances of damage to blood vessels and nerves around the operation corridor. Together, it could be feasible to obtain maximum exposure with the least amount of resection by applying the endoscope-assisted high anterior cervical approach.

## Data Availability

The original contributions presented in the study are included in the article/Supplementary Material, further inquiries can be directed to the corresponding author/s.
